# A Markov chain model of particle deposition in the lung

**DOI:** 10.1038/s41598-020-70171-2

**Published:** 2020-08-11

**Authors:** Adam H. Sonnenberg, Jacob Herrmann, Mark W. Grinstaff, Béla Suki

**Affiliations:** 1 Department of Biomedical Engineering, Boston University, 44 Cummington Street, Boston, MA 02215 USA; 2 Department of Systems Engineering, Boston University, Boston, MA USA; 3 Department of Chemistry, Boston University, Boston, MA USA

**Keywords:** Respiration, Computational models, Statistical physics

## Abstract

Particle deposition in the lung during inhalation is of interest to a wide range of biomedical sciences due to the noninvasive therapeutic route to deliver drugs to the lung and other organs via the blood stream. Before reaching the alveoli, particles must transverse the bifurcating network of airways. Computational fluid mechanical studies are often used to estimate high-fidelity flow patterns through the large conducting airways, but there is a need for reduced-dimensional modeling that enables rapid parameter optimization while accommodating the complete airway network. Here, we introduce a Markov chain model with each state corresponding to an airway segment in which a particle may be located. The local flows and transition probabilities of the Markov chain, verified against computational fluid dynamics simulations, indicate that the independent effects of three fundamental forces (gravity, fluid drag, diffusion) provide a sufficient approximation of overall particle behavior. The model enables fast computation of how different inhalation strategies, called flow policies, determine total particle escape rates and local particle deposition. In a 3-dimensional airway tree model, the optimal flow policy minimizing the risk of deposition at each generation, compared to other inlet flow waveforms, predicted significantly higher probability of escape defined as the fraction of particles exiting the tree. The model also predicts a small influence of body orientation with respect to a gravitational field on total escape probability, but a significant effect of airway narrowing on regional deposition. In summary, this model provides insight into inhalation strategies for targeted drug delivery.

## Introduction

Inhalation of drugs such as bronchodilators to dilate airways in asthmatics is a common treatment modality. The goal is to guide the inhaled drugs through the heterogeneous bifurcating structure of the lung toward the diseased areas. Evaluating the efficiency of a given breathing maneuver requires mapping the spatial distribution of drug deposition in the lung, which is experimentally challenging.

Numerous computational fluid dynamics (CFD) and Monte-Carlo simulation-based models have been proposed to characterize particle deposition pattern in the lung^1–6^, but in most cases, these studies investigated the influence of a single factor such as particle diameter on overall deposition efficiency. Gamma scintigraphy is a useful experimental tool to depict lung deposition for areosols^7^, but generally produces results with lower spatial resolution than CFD. Furthermore, the effects of inhalation flow speed, more precisely flow policy describing optimal flow variation during a breath, on deposition pattern have not been studied. CFD may require long computational times, precluding the investigation of how sensitive the deposition pattern is to wide variations in local geometry or body position. Additionally, the computational burden of CFD also limits the inclusion of both central and small peripheral airways. Several studies also explored stochastic Monte-Carlo approaches to particle deposition^3, 8^ although these models did not consider flow policies or the effects of diseases on deposition. A major benefit of the transition to a Markov model over previous Monte-Carlo studies is the ability to calculate steady state solutions to particle deposition. Previous studies however, only simulated paths of randomly generated geometries often without including proper asymmetry and did not consider all the forces that act upon a particle.

Simulation of particle motion allows the prediction of particle trajectory if all relevant forces are included in the model. The net force ($${F}_{net})$$ acting on a particle in the lungs is the sum of three vectorial forces, namely gravity, drag and diffusion^9^:

1 $$\begin{array}{c}{F}_{net}={F}_{gravity}+{F}_{drag}+{F}_{diffusion} \end{array}$$

These forces contribute to the particle’s probability of escape from or deposition in a given airway. This probability in turn depends on numerous factors such as the length, diameter and orientation of the airway, as well as the flow rate due to breathing or mechanical ventilation. In principle, the probability of escape from a single pipe can be utilized to compute the total probability that a particle traverses the entire airway tree without capture.

The goal of this study was to develop a Markov chain formulation of particle motion in which each state corresponds to an airway segment within which particles may be located. The transition probabilities for a particle to move from one state to another (i.e., from one airway segment to another) incorporate the above three forces. To demonstrate the utility of the approach, we identify optimal inlet flow policies that minimize particle capture in a realistic asymmetric 3-dimensional (3D) tree model of the human airways. This formulation also allows us to determine the effects of body position and airway narrowing on escape and deposition probabilities.

## Methods

In order to turn the deposition problem into an optimization problem, a Markov chain model of particle transport and deposition in a bifurcating structure mimicking the human airway tree was implemented. The Markov chain consists of states connected according to the known bifurcation topology of the airway structure^10^. A particle is considered to be in a particular state when it is located within the corresponding airway segment, either suspended in the fluid flow through the airway lumen or deposited onto the airway wall. The transition probabilities between allowed states are defined below.

Traditionally, the airway tree is labeled as follows. Generation 1 is the root of the tree (e.g., the trachea) and has two daughter generations labeled as 11 and 12 where denoting the left and right daughter branches, respectively. Each parent is connected to only two daughters.

As an example, the scheme in Table 1 replaces the traditional labeling scheme with state numbers in a small 3-generation tree. The mapping of segments into states reveals how the adjacency matrix of the Markov chain is determined. The matrix is sparse and when formulated as a Markov chain allows for rapid simulation (see Supplemental Table S1). A special state is defined at the terminal ends of the tree representing the terminal bronchioles, beyond which more bifurcations are not considered. When a particle reaches this state, it stays there afterwards with probability 1. The formulation of the transition probabilities, the probability of changing from one state to another, requires the establishment of the probability that a particle deposits by sedimentation, impaction, or diffusion, which is considered next.

**Table 1:** Mapping of standard airway labeling to Markov chain states.

Airway segment	Markov state
1	1
11	2
12	3
111	4
112	5
121	6
122	7

### The probability of deposition due to gravitational sedimentation

The sedimentation probability (*p*_*s*_) is defined as the probability that a particle will deposit by gravity. For Poiseuille flow in a cylindrical tube of length *L*, diameter *D*, having an angle $\theta$ with respect to the horizontal direction (gravity is vertical), a simplification^11, 12^, and a generally accepted method of estimating *p*_*s*_ can be used if the particle diameter (*d*_*p*_) is small defined as

$${d}_{p}\ll {d}_{s} \hspace{5mm} where \hspace{5mm}{d}_{s}=\sqrt{\frac{72Q\mu }{sin\theta {\rho }_{p}g\pi {D}^{2}}}$$

where *Q* is the actual flow through the tube, ${\rho }_{p}$ is the density of the particle, *g* is the gravitational acceleration and $\mu$ is the viscosity of the fluid. Let us introduce the following quantity:

$$\kappa =\frac{3}{4}\frac{{v}_{s}}{\left(\frac{Q}{\pi {\left(\frac{D}{2}\right)}^{2}}\right)}\frac{L}{D}cos\theta$$

where ${v}_{s}$ is the settling velocity of the particle. This expression is approximately equal to the ratio of the time required for a particle to axially travel through the tube to the time to reach the wall by settling. The *p*_*s*_ can now be written as

2 $$\begin{array}{c}{p}_{s}=\frac{2}{\pi }\left[2\kappa \left(\sqrt{1-{\kappa }^\frac{2}{3}}\right)-{\kappa }^\frac{1}{3}\sqrt{1-{\kappa }^\frac{2}{3}}+arcsin\left({\kappa }^\frac{1}{3}\right)\right] \end{array}$$

Notice that if $\theta =-\pi /2$, i.e., the tube’s axis is parallel to gravity, $\kappa =0$ and the probability of capture by sedimentation is zero.

It is worth noting that as the Reynolds number, defined as $Re=\frac{4\rho Q}{\pi D\mu }$ (where $\rho$ is fluid density), and/or the Stokes number (defined below) increase, particles move quicker and $\kappa \to 0$ so that the probability that a particle deposits due to sedimentation approaches zero^13^. Particles transverse the tube before they can deposit. Thus, for high-speed flows, ${p}_{s}$ is effectively zero.

### The probability of deposition due to impaction

A particle’s deviation from a fluid stream line is due to the relaxation time, and thus its probability of impaction can solely be determined by the Stokes number^13, 14^. The Stokes number is defined as

3 $$\begin{array}{c}Stk=\frac{{U}_{0}{\rho }_{p}{{d}_{p}}^{2}{C}_{c}}{18\mu D}\end{array}$$

where ${U}_{0}$ is the local fluid velocity and ${C}_{c}$ is the Cunningham slip correction factor. The probability of impaction ${(p}_{i})$ can be calculated using the Stokes number. For small values of Stk, the following simplification can be applied^15^:

4 $$\begin{array}{c}{p}_{i}=1.606Stk+0.0023\end{array}$$

When analyzing the influence of Stk on impaction across a wide range, a saturation phenomenon occurs that can be modeled using a sigmoidal function^13^. In preliminary studies, we found that values of Stk for the present study were below 0.15 and fell within the linear portion of the sigmoidal. Hence, Eq. 4 was used in all subsequent analyses. When $Stk$ is very low corresponding to low-speed flow or very small particle diameter, the particles tend not to impact, resulting in negligible contribution of impaction to the probability of deposition by impaction. It is interesting to note that for micron-sized particles in a given flow, the sedimentation and impaction probabilities are nearly inversely proportional.

### The probability of deposition due to diffusion

Generally, diffusion is considered to be the least important of the three forces contributing to particle deposition, especially when considering particles of larger diameters (> 1 µm). However, for particles in the nanometer size range, deposition by diffusion can be significant. Following a previous derivation^11, 16^, we first introduce the following variable:

5 $$\Delta =\frac{{k}_{B}T{C}_{c}}{3\pi \mu {d}_{p}}\frac{L}{\stackrel{-}{U}}\frac{1}{{4 \left (\frac{D}{2}\right)}^{2}}$$

where ${k}_{B}$ is the Boltzmann constant, *T* is absolute temperature and $\stackrel{-}{U}$ is the mean fluid velocity. Equation 5 is the coefficient of diffusion of the particle non-dimensionalized by the properties of the bulk flow. For Poiseuille flow, Eq. 5 is then used in the expression of the probability of diffusion-induced deposition:

6 $${p}_{d}=1-0.819{e}^{-14.63\Delta }-0.0967{e}^{-89.22\Delta }-0.0325{e}^{-228\Delta }-0.0509{e}^{-125.9{\Delta }^\frac{2}{3}}$$

$$\text{if}\hspace{5mm} \Delta <0.16853$$

and $${p}_{d}=1\hspace{5mm} \text{if} \hspace{5mm}\Delta \ge 0.16853$$.

Similar to the relationship between low flow speeds and increased chance of gravitational sedimentation, low flow speeds are also associated with increased chance of diffusion-induced deposition. The relative contribution of diffusion to overall particle motion is greatest during low-flow conditions, when the particle traverses an airway slowly and thus has more time to diffuse radially and deposit. However unlike sedimentation, diffusion is the result of random motion, and therefore is not affected by tube orientation within the gravitational field.

It is important to note that various other probability formulations exist^13, 15^; indeed, *The Mechanics of Inhaled Pharmaceutical Aerosols*^11^ lists over 10 formulations for inertial impaction alone. It is not the purpose of this study to introduce new formulations. In essence, they all reduce to an inverse relationship between particle deposition by impaction vs. deposition by diffusion or sedimentation. High flow rates are associated with greater contributions of impaction, whereas low flow rates are associated with greater contributions of diffusion and sedimentation. Finding the interplay between these conditions and formulating particle transport as a Markov chain allows us to obtain insight into optimal drug delivery strategies.

### Adjacency matrix for realistic geometries

All Markov chains require an adjacency matrix to define the probability of transition from one state to another. To generate the adjacency matrix, two special states are created. The second to last column in the Supplemental Table S1 represents the probability that a particle is captured in an airway segment anywhere in the tree, and a special terminal escape state is introduced in the final column representing the alveolar space. The total escape probability (${p}_{e}$) that a particle transitions from a given airway segment to either of its daughter segments is calculated by combining the individual escape probabilities for each of the three forces as follows:

7 $$\begin{array}{c}{p}_{e}=\left(1-{p}_{s}\right)\left(1-{p}_{i}\right)\left(1-{p}_{d}\right)\end{array}$$

Equation 7 states that the probability that a particle is not captured in a segment is the product of 3 probabilities each describing the probability of escape by one of the 3 forces. It is thus the chance of not being captured by gravity, diffusion or impaction. In this formulation we assume that these probabilities are independent as in previous studies^3, 8, 17^. This probability is then partitioned between the two daughter branches based on the expected flow division that can be obtained using either the downward pathway resistance of the two daughter branches^18^, or CFD simulations (see below). For the former case, the downstream input resistances of both subtended trees of a given segment, including each daughter and all subsequent granddaughters, were computed from the tree geometry assuming normal air and Poiseuille flow. Considering that the two daughter subtrees are in parallel, the flow partitioning between the two daughters is such that the flow entering a subtree is inversely proportional to the subtree’s total input resistance. The probability of a particle transitioning to one of the daughter branches is then obtained by multiplying Eq. 7 by the fractional flow into that segment. In this way, the fractional flow in one daughter branch determines the relative probability of particle transition to that branch compared to the other daughter. Once a particle is captured in a segment or reaches the terminal state, it remains there. This is achieved by allowing these states in the Markov chain to be “sticky,” i.e., they communicate to themselves with a probability of 1.

The full transition matrix for a 3-generation simple tree is given in Supplemental Table S1. Note that for the terminal generations, there are no bifurcations at the end, and they are assigned a non-zero probability based on Eq. 7 to transition to the terminal escape state. The above method of adjacency matrix generation was applied to the 3-dimensional airway^10^ that includes segments down to the terminal bronchioles. Relevant geometric data (i.e., lengths, diameters and angles) were imported and used to compute the transition probabilities according to Eqs. 2–7.

### Computational studies

Computations including the creation of geometries and the Markov chains, were carried out using MATLAB (R18b, Mathworks, MA). For all simulations, particles were also assumed to have the density of water and the temperature was set to 37 °C.

To provide evidence for the validity of Eq. 7, we set up a CFD model of a single bifurcation of a main human bronchus, simulated over a wide range of inlet flow rates representative of human breathing conditions. The bifurcation model was longitudinally extended to account for flow development, such that the flow inlet of the model preceded the entrance of the parent airway segment by 20 times. The inlet flow was assigned a uniform velocity profile. The velocity field was solved in Comsol Multiphysics 5.4a for different Reynolds numbers. Large eddy simulation (LES) using the Smagorinsky sub-grid scale model was used in accordance with previous computational experiments^19^. Two spatial distributions of particles were simulated at the entrance of the parent airway for the particle transport simulations: a uniform distribution over the airway cross-section, and a parabolic distribution with particle concentration highest at center of the airway. Simulated particles were then released on top of the fluid flow solution with their motion governed by Newton’s second law. Modeled forces in the particle transport simulation included the drag force from the fluid flow solution, gravity, and diffusion. Total probability of particle capture was computed using the number of particles depositing within the parent airway or at the bifurcation. Further details regarding mesh generation and fluid simulation approach can be found in the Supplemental material.

The above adjacency matrix formulation provides a simple way to determine optimal flow policies. For example, if the goal is to target delivery to the alveoli beyond the terminal airways, the optimal flow policy should maximize particle escape from the entire tree. To achieve this, we first consider a single tube, for which the total probability of capture (${p}_{c}=1-{p}_{e}$) must be minimized with respect to the inlet flow waveform:

8 $$\begin{array}{c}{Q}^{*}=\frac{min}{Q\in Q\left(x\right)} {p}_{c}\left(Q\right)\end{array}$$

where $Q(x)$ is the set of physiologically admissible flows into that segment and ${Q}^{*}$ is the optimal flow. In the perfect state problem for which the geometry of the system and the velocities and locations of all particles are known, the only controllable variable to consider is *Q*. All other properties of the particle and the geometry are assumed fixed. Additionally, ${p}_{s},\,{p}_{i},\, \text{and} \,{p}_{d}$ are all functions of the flow rate and thus, taking the derivative of the total probability deposition with respect to *Q* and setting it equal to zero gives:

9 $$\begin{array}{c}\frac{d}{dQ}\left(1-\left[\left({1-p}_{s}\right)\left(1-{p}_{i}\right)\left(1-{p}_{d}\right)\right]\right)=0\end{array}$$

Equation 9 allows solving for a minimum that would be the candidate for an optimal flow rate ${Q}^{*}$ for a single tube.

**Figure 1:**
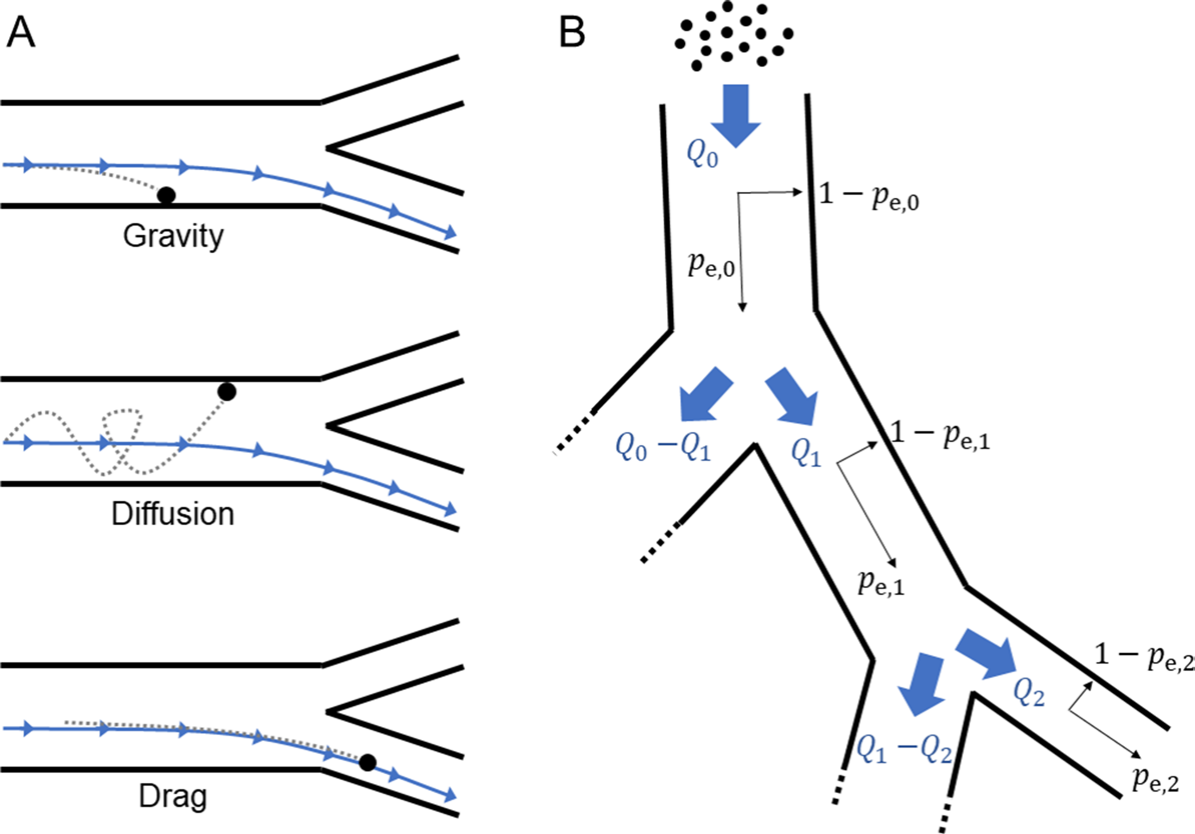
(**A**) Schematic representation of the fundamental forces—gravity, drag, and diffusion—which determine particle motion and capture in this model. (**B**) A general schematic for the flow division at bifurcations. The ${p}_{e,0}$ and ${p}_{e,1}$ are the escape probabilities from the 0th and 1st airway segments, which depend explicitly on the instantaneous flow through those segments as given by Eq. 7.

An optimal flow policy providing the global minimum for particle capture in the entire tree may be obtained by considering all segments. In a symmetric tree with symmetric flow bifurcations, this problem reduces to finding the optimal flow minimizing Eq. 7 at each generation, since the flow rates in all airways of any particular generation may be assumed homogeneous. In this way, the entire optimization problem is deconstructed to the individual airway segment level. The particle path is considered as a cascade of individual bifurcating units using a formulation similar to that reported by de Vasconcelos et al.^13^. However, it is worth noting that their work did not include the influence of gravity, nor did it consider flow policy or an asymmetric geometry. By contrast, finding the optimal flow policy for an asymmetric tree is complicated by the fact that the capture probabilities cannot be independently calculated for each generation, due to asymmetric flow partitioning at bifurcations. Instead, this problem formulation must consider the capture probabilities in all airway segments along each particular path from trachea to periphery. A general equation for describing particle escape in asymmetric geometry can be obtained as follows. The formalism describing the optimal flow is related to the “path escape” probability (${P}_{e}$) of a single particle escaping the tree along a particular path from the trachea to a terminal segment:

10 $${P}_{e}={p}_{e,0}\left({Q}_{0}\right)*\left[\frac{{Q}_{1}}{{Q}_{0}}{p}_{e,1}\left({Q}_{1}\right)\right]*\left[\frac{{Q}_{2}}{{Q}_{1}}{p}_{e,2}\left({Q}_{2}\right)\right]*\dots *\left[\frac{{Q}_{n}}{{Q}_{n-1}}{p}_{e,n}\left({Q}_{n}\right)\right]=\frac{{Q}_{n}}{{Q}_{0}}\prod_{k=0}^{n}{p}_{e,k}({Q}_{k})$$

where $n$ is the number of airways segments along the path and ${p}_{e,k}$ is the escape probability for the $k$th airway segment along the path, which depends explicitly on the instantaneous flow ${Q}_{k}$ through that segment. A visual representation of Eq. 10 is given in Fig. 1. The product includes all segment escape probabilities weighed by their fractional flows along the path connecting the root inlet flow ${Q}_{0}$ to the terminal end of the tree flow ${Q}_{n}$.

Notice that the fraction in front of the product on the right hand side is the fraction of flow going through the terminal segment. This probability is very small; however, when we sum the probabilities for all ${N}_{t}$ terminal segments, we obtain the total probability of escaping the tree (${\mathbb{P}}_{e}$):

11 $$\begin{array}{c}{\mathbb{P}}_{e}\left({Q}_{0}\right)=\sum_{i=1}^{{N}_{t}}{P}_{e,i}\end{array}$$

This probability is the sum over all unique ${N}_{t}$ paths of the individual path escape probabilities in Eq. 10. Furthermore, if the inlet flow ${Q}_{0}$ varies in time, the optimal time-varying flow waveform must account for particle positions at each consecutive time point, and update all downstream single-tube escape probabilities. Since we neglect turbulence in the flow calculations, the flow partitioning is computed only once. Updating the flow rates throughout the tree for a time-varying problem requires simply scaling each flow rate in proportion to changes in ${Q}_{0}$. This allows quick calculation of the capture probabilities and hence optimization of a time-varying inlet flow profile for maximal escape from the tree. Thus, Eqs. 10 and 11 were used to compute the probability of escape as well as regional deposition.

To compare the Markov chain model to experimental results, we mimicked the data produced by gamma scintigraphy as follows. A 3D grid of voxels with isotropic 5 mm spatial resolution was superimposed on the model geometry. Each voxel within an airway segment was assigned a deposition probability value equal to the deposition probability for that airway segment multiplied by the ratio of voxel volume to segment volume. Particles escaping a terminal airway segment were represented by a uniform probability distribution within a 3 × 3 × 3 cube of voxels at the distal end of the terminal airway. The 3D probability maps were then integrated along the ventral-dorsal axis to obtain a 2D coronal projection, such that each pixel value represented the deposition probability per unit area of the projection.

The effects of alterations in geometry and the sensitivity of the solutions were explored as follows. The effects of changing the orientation of the tree was determined. Since each branch of the tree is specified by the start and end point in 3D space, the entire tree was rotated by multiplying each branch with a 3D rotation matrix. Rotation around the vertical axis caused no change in the overall deposition pattern. Thus, only rotations relative to gravity were simulated to investigate the effects of body position on deposition pattern.

## Results

**Figure 2:**
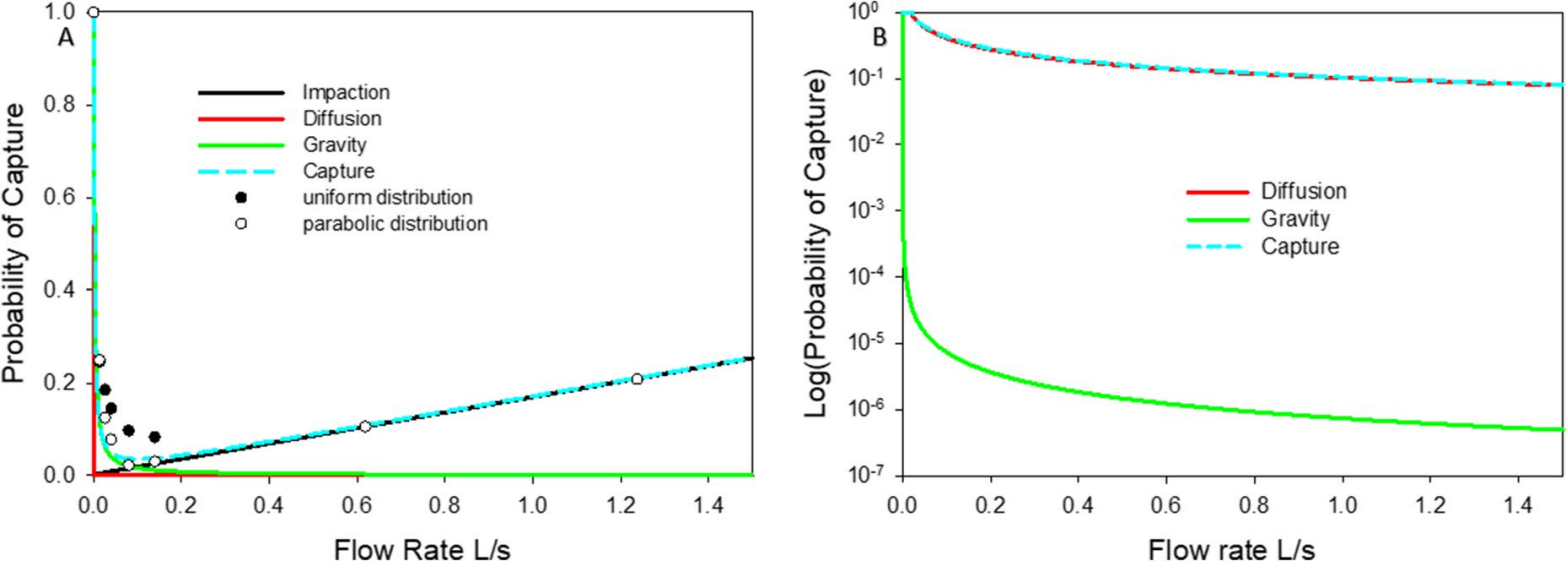
Probability of capture due to gravity, impaction, diffusion and all three combined in a tube with dimensions corresponding to a third generation bifurcation (length = 6.3 cm, diameter = 1.35 cm, dry air) with its axis perpendicular to gravity. Panel (**A**) and (**B**) show results for a large particle and a small nanoparticle with diameters of 8 μm and 0.35 μm, respectively. Comsol-based computational fluid dynamics (CFD) simulation of escape rate is also shown in panel A for either a uniform distribution (black circle) or parabolic distribution (white circle) for particles released at the airway entrance. Note that the impaction probability for the nanoparticle is virtually zero and not plotted on panel (**B**).

First, we investigated the relationship among the 3 forces in a single tube corresponding to the human main bronchus. Flow rates between 0.0001 and 1 L/s were tested, which represents the full range of human flow rates. Figure 2 compares the probability of capture—computed as 1 minus the probability of escape in Eq. 7—due to gravity, impaction and diffusion for a large 8 μm diameter particle (panel A) and a small 350 nm diameter nanoparticle (panel B). For the large particle (panel A), deposition by diffusion is negligible as expected. Deposition due to gravity decreases while deposition due to impaction increases with inlet flow rate. The combined effects of all three forces (Eq. 7) lead to a tradeoff with a minimum of the overall probability at around 0.2 L/s for the large particle. The total capture rates from CFD simulations are in reasonable agreement with those predicted by Eq. 7 which assumes independent effects of drag, gravity and diffusion on particle motion. The sum of squared differences between our model and the CFD simulations was reduced by 53% using a parabolic distribution of particle inlet velocities at the airway entrance compared to a uniform distribution. For the small particle (Fig. 2B), we find a monotonous decrease of the capture probability with increasing flow rate. The angle in these simulations was 0 degrees which means the tube’s axis is perpendicular to the direction of gravity. For the nanoparticle, the influence of diffusion is dominant at all flow rates, while gravity is only important close to no flow, and impaction disappears. The probability of capture by diffusion is also not affected by the orientation of the tube.

The asymmetric geometry of the human airways containing up to 23 generations was next transformed into a Markov chain model. Before carrying out simulations for different inlet flow profiles, we first validated our Markov chain-based particle deposition in the airway tree. Specifically, we plotted digitized existing experimental data^20^ against the deposition predicted from our Markov model.

**Figure 3:**
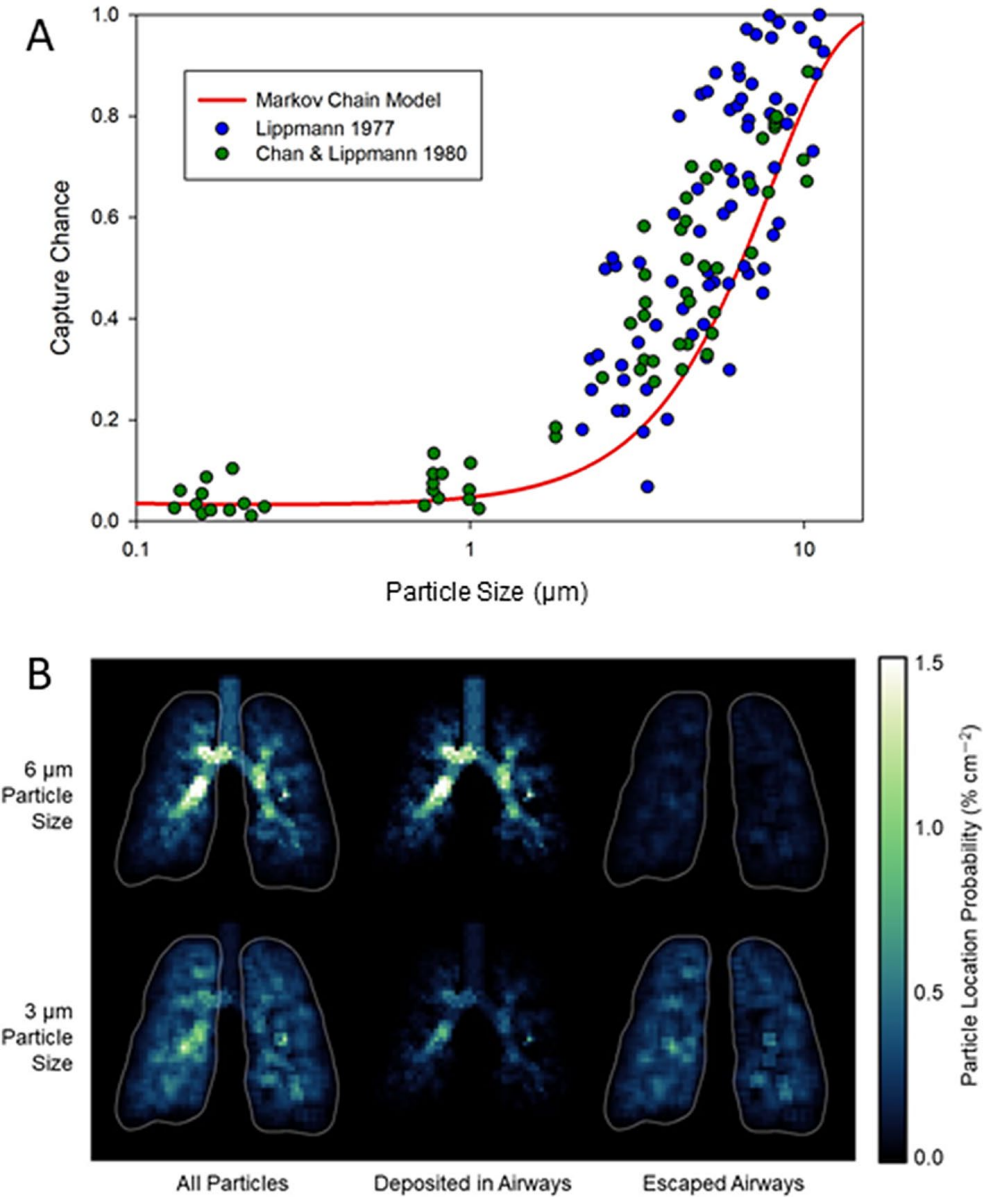
(**A**) Comparison of the Markov chain model with existing experimental data. Our simulations were conducted for a fixed flow rates of 0.5 L/s which was the same as in the studies plotted. Probability of capture was plotted as a function of particle size. (**B**) Coronal projections of the 3D deposition and escape probabilities obtained from the Markov chain model, for comparison with gamma scintigraphy.

Previous studies have used this data set to show agreement with various computational results^3, 17, 21, 22^. Figure 3A demonstrates that the total capture rates from the literature are in reasonable agreement with those predicted by Eq. 10 which assumes independent effects of drag, gravity and diffusion on particle motion. Additionally, as demonstrated in Fig. 3B, our predicted 2D projections of deposition probability are in good qualitative agreement with experimental data obtained using Krypton ventilation scan and gamma scintigraphic lung images^7^ since the predicted total, pure airway and peripheral deposition (escape probabilities), fall within collected experimental data ranges (see Fig. 1 in Ref. 7).

**Figure 4:**
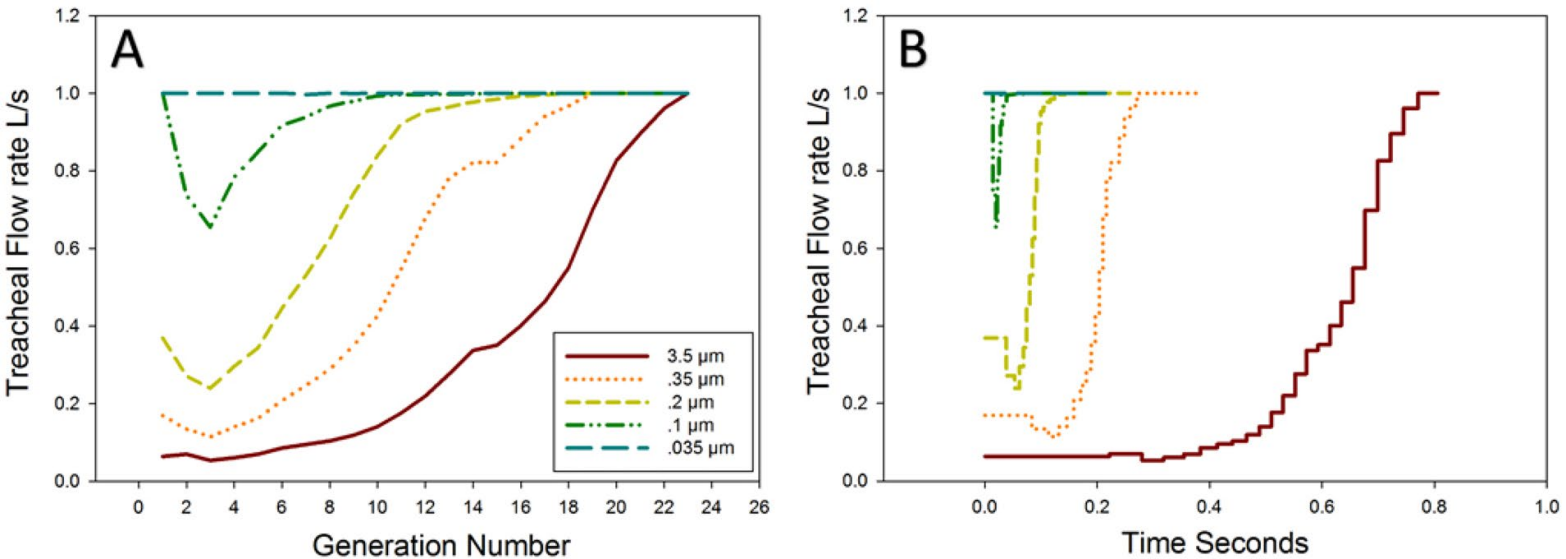
(**A**) Optimal inlet flow rates at the trachea minimizing capture at each generation of the human airway geometry using a 3-dimensional airway tree for several particles sizes. The first generation is the trachea and since it is orientated vertically the flow that minimizes deposition is low. As generation number increases the risk of deposition due to inertial impaction for a given particle decreases and hence optimal flow increases. (**B**) Optimal flows as a function of time. Note that the time of the flow only represents a window during inspiration (see text for further explanation).

We next identified the inlet flow waveform, or flow policy, that maximizes the number of transitions before deposition. For every generation, this was accomplished by finding the minimum of Eq. 9. The average optimal inlet flow rate at the trachea (i.e., minimizing the probability of capture at each generation) is plotted in Fig. 4A for particle sizes covering 2 orders of magnitude. The corresponding time-varying flow policy is shown on Fig. 4B. It is important to note that Fig. 4B does not show the entire inspiratory waveform, but rather only the interval during which a single particle is expected to escape the tree starting from the trachea. Thus, particle release is not assumed to coincide with the beginning of inspiration, and a non-zero initial flow rate is an admissible flow policy. For example, the optimal policy for a particle of size 0.035 μm is a short 0.2 s burst of maximum flow at the trachea. This means that the inspiratory flow waveform should contain a 0.2 s interval with 1 L/s flow rate and the particles should be released at the trachea at the beginning of this interval. In general, for particles larger than 0.1 μm, optimal time-varying flow policy applies increasing inlet flow rate as particles transition to more peripheral airway segments.

**Figure 5:**
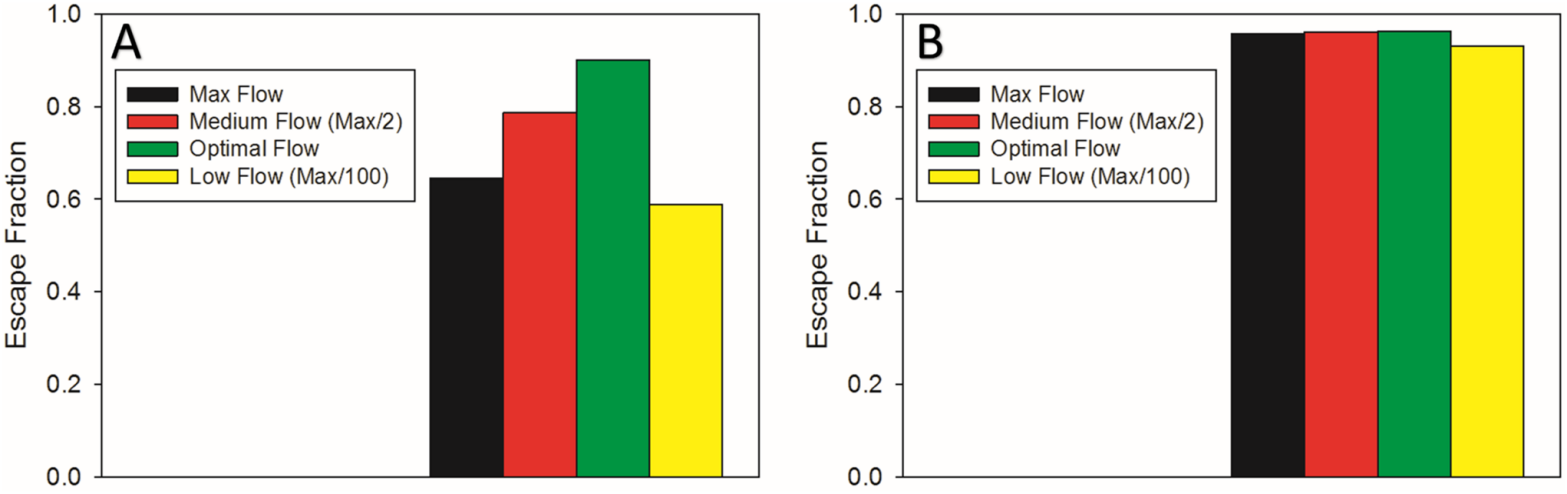
Probability of escape of the tree computed for 4 different flow policies. The optimal policy is the time varying flow rate identified in Fig. 4. Panels (**A**) and (**B**) show the probability of escape for the 3.5 μm and the 0.35 μm particles, respectively.

**Figure 6:**
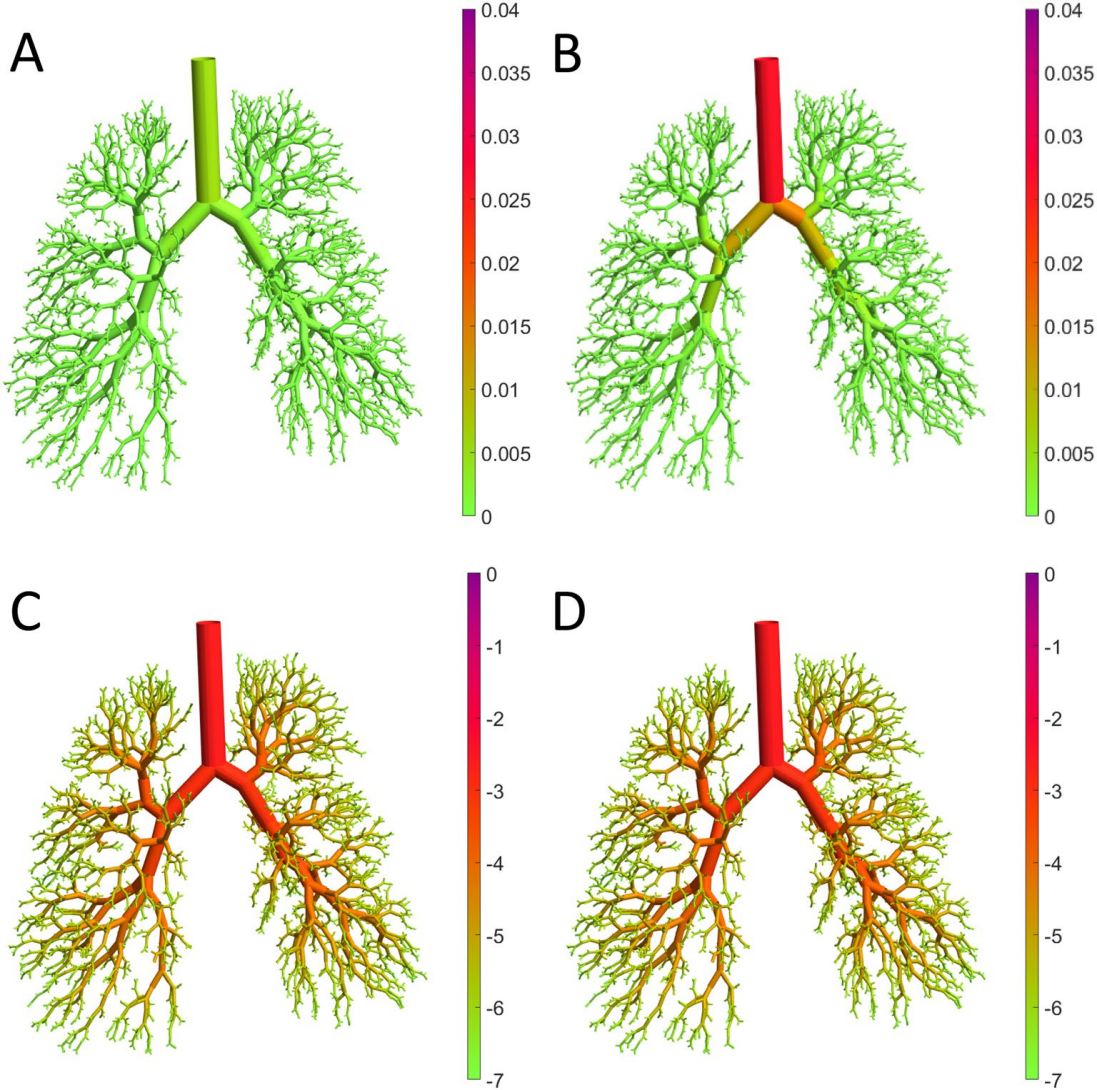
Steady state solutions of the probability of capture for optimal flow policy (**A**) and maximum flow policy (**B**) using the 3.5 μm particle. Probability of capture for the 0.35 μm for the optimal flow policy and for a maximum flow are shown in (**C**) and (**D**), respectively. Color intensity is proportional to the probability for a given segment for panels (**A**) and (**B**) and, because of the wide range of values, the logarithm of probability for panels (**C**) and (**D**).

To test the effects of different flow policies on the probability of escape defined as the fraction of particles delivered to the preiphery of the tree (Eq. 11), simulation were carried out (Fig. 5) using a 3.5 μm diameter particle (panel A) and the 0.35 μm diameter nanoparticle (panel B). The maximum flow rate is defined as a constant inlet velocity at the highest possible flow rate. The results suggest that with the larger particle, the time-varying optimal flow policy from Fig. 4B is significantly more efficient with probability of escape of ~ 0.24 higher than the maximum flow rate using constant flows. For the nanoparticle, low inhalation flow rates are worse than the optimal policy. However, the optimal flow policy converges to the maximum flow rate since the risk of impaction is nearly zero. The spatial distribution of particle deposition was obtained by visualizing the probability of capture for the optimal flow policy and the maximum flow for both particle sizes (Fig. 6). The color intensity represents the steady state solution of the Markov chain model. For the 3.5 μm particle, the maximum flow led to significantly more deposition throughout the tree. However, as expected, there was little difference in deposition between optimal and maximum flow policies in the case of the 0.035 μm particle. However, in the presence of a narrowing, mimicking for example airway constriction, the maximum flow policy is no longer efficient. Indeed, constriction of a single segment significantly alters both flow and deposition (see Supplemental Fig. S2).

**Figure 7:**
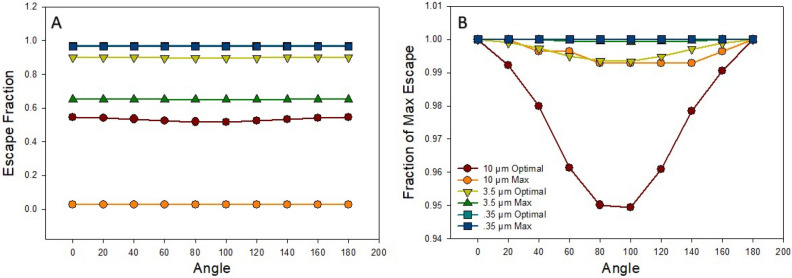
(**A**) Probability of escape of particles plotted against the angle of rotation for the proposed optimal flow policy waveform and maximum flow waveform for several particle sizes. (**B**) The corresponding percent of maximum escape for all particles and flows as a function of angle.

Figure 7A shows how the total escape probability depends on body orientation with respect to gravity for various sized particles. Total escape probability was much more sensitive to particle size than body orientation. Figure 7B demonstrates the variations in relative terms: there was a 5% difference in escape for the largest particle between horizontal and vertical orientations.

## Discussion

In this study, we introduced a novel approach to study deposition in a 3D airway tree, and used it to determine the optimal flow policy that maximizes delivery at the terminal ends. The model is probabilistic and based on the concept of a Markov chain which requires an adjacency matrix with transition probabilities computed from the physics associated with the motion of particles along prescribed flows in rigid tubes. The model, together with our formulation of path escape and total escape probabilities, allows quick calculations of total and regional probabilities for particle deposition or escape throughout the entire tree. The main findings are that (1) the optimal flow policy provides the highest probability of particle delivery to the lung periphery, especially for larger particles; (2) for nanoparticles there is negligible impact of varying flow policy on peripheral particle delivery; (3) orientation of the tree with respect to gravity makes little difference in terms of escape rate, at least compared to the influence of particle size; and (4) a single airway closure significantly alters flow and deposition patterns.

To explain these results, we note that in the first few generations, for particles in the micron size range, the risk of depositing by inertial impaction is significantly greater than by sedimentation or diffusion. Additionally, the trachea descends in the direction of gravity, and thus there is little penalty against escape for slowly traversing particles because there are no boundaries for sedimentation. In any segment that points in the direction of gravity, the risk of gravitational sedimentation is low, and thus moving too slowly carries little risk of deposition. Nevertheless, we cannot generalize from this finding since airways within the same generation may possess a wide range of angles.

As the micron-sized particles move deeper into the airway tree, the flow rate decreases faster than the diameter, and thus larger flow rates can be applied without the risk of inertial impaction.Thus, a ramp-up policy involving low flow rates in the beginning while particles traverse the first few generations, followed by increasing flow rates as particles traverse the peripheral airways, appears to maximize the probability of escape into the alveolar space. However, certain airways possess lower optimal flow rates than other airways of the same generation number, depending on alignment with the gravitational field.

The optimal flow policy for nanoparticles is slightly different. The risk of depositing by inertial impaction is nearly zero, since nanoparticles do not move fast enough to raise the Stokes number above one. Consequently, there is no reduction in the probability of escape when applying high flow rates throughout the airway tree. However, applying low flow rates reduces the probability of escape (Figs. 2A and 5B) as particles are captured due to diffusive forces. This is an important finding since current clinical practice is to inhale drugs slowly. Additionally, diffusion is unaffected by the orientation of the airways. Thus, body position does not matter much with respect to nanoparticles (Fig. 7) .The optimal policy for particle escape is to use the maximum flow rate throughout the delivery window during inspiration, to deposit particles as deep as possible into the airway tree. This partially explains why the maximum flow simulation and the optimal waveform exhibit similar escape probabilities (Fig. 5B).

It is important to note that the optimal flow policies for the two different particles are not identical and hence the corresponding deposition patterns cannot be directly compared. Nevertheless, we can compare the deposition patterns corresponding to the maximum flow policy for the 3.5 µm (Fig. 6B) and the optimal flow policy for 350 nm particle (Fig. 6C) because the optimal policy for the latter is virtually identical to the maximum flow policy. It is evident that more micron-sized particles deposit in the airways than nanoparticles which have higher escape probabilities (Fig. 5) in agreement with experimental observations^23^. Larger particles deposit earlier than the nanoparticles and thus were found to have a quicker clearance time. Indeed, the maximum flow policy for the micron-sized particle produces a probability of escape < 0.65 (Fig. 5A, black) whereas optimal flow policy reaches a probability of escape > 0.9.

The finding that the orientation relative to gravity had little effect on escape (Fig. 7) may appear surprising. However, this makes sense if we consider that it is only the gravitational sedimentation that is dependent on the angle of the tube. After several generations the distribution of the orientations for the tubes becomes approximately uniform and hence no specific orientation is favored.

To extend our approach to the optimization of particle deposition to a specific region of the airway tree, one only needs to use Eq. 10 and not sum over all paths. Once the particle reaches the desired region without deposition, the flow can be stopped so that the drag force disappears and the particle settles by diffusion or gravity. In short, this requires minimizing the capture chance of the path before the desired segment and maximizing the capture chance by a breath hold once the particle arrives to the desired segment. For example, a flow policy for targeting the third generation of a tree involves minimizing deposition in the first two generations and then applying a breath hold maneuver to facilitate particle deposition. To our knowledge, constructing such optimal flow policies for maximum delivery has not been proposed to date.

Our CFD results reveal an interesting discrepancy between the assumptions underlying the gravitational sedimentation model and the reported outcomes (Fig. 2). CFD results for particle capture were in better quantitative agreement with the Markov model when a nonuniform parabolic distribution of particles was simulated at the airway entrance, as opposed to a uniform distribution. By contrast, the experimental and modeling work from which the gravitational sedimentation model was obtained, assumed a uniform particle distribution at the inlet^12^. However it is plausible that gravitational settling and Taylor dispersion resulted in non-uniform particle distributions at the entrance of the experimental model, due to the absence of an aerosol mixer near the inlet sensor. This could explain why the parabolic inlet condition corresponded better with the experimental results than the uniform distribution.

**Figure 8:**
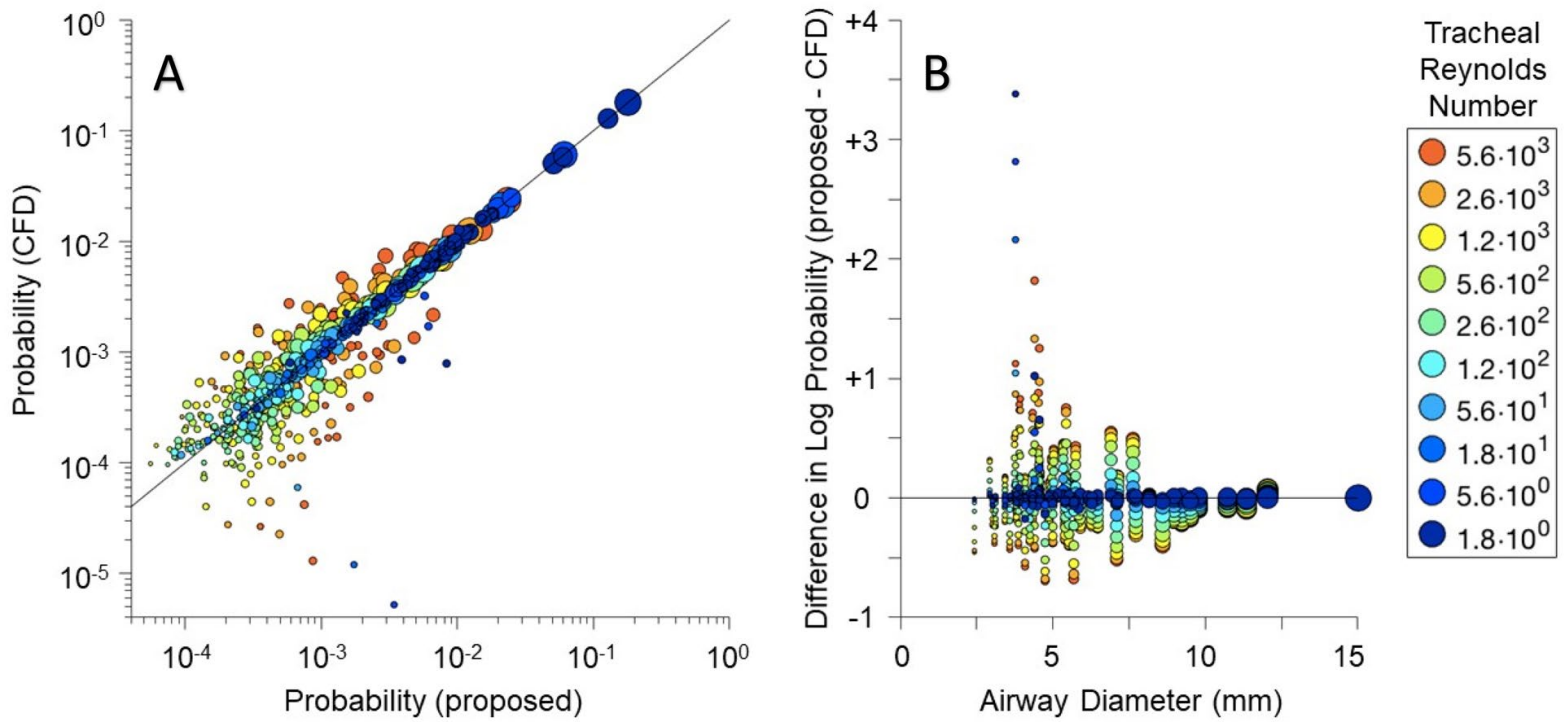
The linear relationship between predicted probabilities for a 3.5 micron sized particle using either a computational fluid dynamics (CFD) simulation or a resistance-based approach (proposed) is shown in (**A**). Each circle represents particle capture probabilities for an airway segment, with size indicating airway diameter and color indicating tracheal Re. The solid line represents identical probability predictions by each model. The relationship between discrepant probability predictions and airway diameter is shown in (**B**).

There are several limitations of the approach presented here. For example, even in a symmetric bifurcating structure, flows are not symmetric at higher Reynolds numbers^24^ when the flow is inertia-dominated. This makes the distribution of particles non-uniform within cross-sections in the central airways, but the Reynolds number drops significantly past the first few generations. An important assumption of the model was that flow partitioning can be obtained using linear resistance approximations for individual airway segments. To examine how a more realistic flow distribution alters the deposition pattern, we solved the spatial distribution of flow with CFD for the upper part of the tree containing 67 segments for a wide range of Re values. More detail to the methods for generating these results are given in the Supplemental material. The flows and the capture probabilities are given in Supplemental Figs. S5 and S7 whereas the correlation between CFD and resistance-based captures is shown Fig. 8.

There is a relatively good agreement between the particle depositions predicted by the two methods. Although there does appear to be large discrepancies between resistance-based and CFD predictions of relative flow, particularly in the smaller distal airways at high Reynolds numbers (Fig. S6), the overall impact on particle deposition is minor (Fig. 8). Discrepancies between the CFD and resistance-based predictions appear to depend most strongly on inertial tendencies of flow, i.e., the resistance-based model does not account for bifurcation angle nor the tendency for flow to distribute preferentially down aligned pathways with minimal change in direction. This likely explains the larger discrepancies at higher flow rates in more peripheral airway segments, due to compounded errors after multiple bifurcations. Nonetheless the Markov chain does an excellent job of predicting overall distributions of particle deposition, especially in the larger central airways at lower Re, and with a much lower order of model complexity.

The linear nature of the inertial impaction term (Eq. 4) was also explored as a potential limitation. An alternative model was implemented for a sigmoidal model of impaction^13^ and its effects were investigated on the probability of impaction in a limited set of simulations. For all Stokes numbers encountered in these simulations, the impaction probability term fell on the linear part of the sigmoidal curve and hence was an acceptable approximation for particles under 10 µm in size. Larger sized particles would require implementation of a sigmoidal impaction; however, the linear model was included in this study as it is a good representation of average collected experimental data for physiologically relevant inertial impaction^11^. Thus, we conclude that for bifurcating cylindrical geometries with angles similar to those in the human lung, human flow rates, and particles smaller than 10 µm in diameter, the current model can be used with reasonable accuracy.

We also did not consider the irregular geometry of the upper airways. It is conceivable that the upper airways would significantly alter the optimal flow policy. Thus, the optimal flow policies we suggest are only for flow in the central and lower airways. Furthermore, the optimal flow policy considered only the independent optimization of generational segments. In an asymmetric tree and in the presence of turbulence and disease this may not provide the best flow policy and a global optimization including all segments simultaneously would be needed. We also neglected the fact that airway dimensions change during inspiration although this is not expected to significantly influence the conclusions. Additionally, flow partitioning did not take into account any differences in regional compliances at the end of the tree. Finally, the approach cannot provide exact locations for individual particles, only the probability of where they deposit. Therefore, the trajectory of a particle does not cascade from one generation to the next. The assumption in the model is that every branch starts with a uniform distribution of particles. As particles transverse from a randomly orientated tube through a sequence of subtended randomly orientated tubes, the effects of cascading is expected to average out.

## Conclusion

We introduced a novel discrete time Markov chain model that simulates particle deposition in a realistic 3D model of the airway tree. We also propose a general formulation for calculating capture chance for any given segment in the airway structure. For a given flow policy, the probability that particles escape the airway tree and deposit in the alveoli was computed. As such this model, which is facile to implement and flexible to accommodate new features, provides an efficient tool for studying deposition to gain insight into ventilation strategies for targeted drug delivery in the healthy and diseased lung.

## Supplementary information

Supplementary Information.
