# Supplementary material for: A Markov chain model of particle deposition in the lung
Source: Sci Rep. 2020 Aug 11;10:13573. doi: 10.1038/s41598-020-70171-2 (PMC7419522; doi:10.1038/s41598-020-70171-2)
Supplement: Supplementary file 1 — Supplementary Information. [file 41598_2020_70171_MOESM1_ESM.docx]

Title

- A Markov chain model of particle deposition in the lung
- Supplementary Information

**Authors**

Adam Sonnenberg^1,2^, Jacob Herrmann^1^, Mark W. Grinstaff^1,3^, Béla Suki^1,^*

**Affiliation**

^1^Department of Biomedical Engineering, Boston University, Boston, MA, USA

^2^Department of Systems Engineering, Boston University, Boston, MA, USA

^3^Department of Chemistry, Boston University, Boston, MA, USA

*** Correspondence:**

Béla Suki

[bsuki@bu.edu](mailto:bsuki@bu.edu)

Department of Biomedical Engineering,

Boston University

44 Cummington street,

Boston, MA 02215, USA

Tel: 1 617 353-5907

***Mesh and computational description: single bifurcation***

The geometry used to study the CFD and particle transport problem was created in Solidworks using a series of circles on defined planes. The bifurcation was generated using a lofted sweep and a fixed radius of curvature of 5.4 times the radius of the parent segment. The Carinal radius of curvature was set to 0.1 times the diameter of the daughter. The bifurcation angle was set to 90 degrees. This solid was next imported directly to COMSOL Multiphysics 5.4.0.388 where a free tetrahedral mesh was created. The number of boundary layers was set to 3. The geometry was appended with an additional tube added to the parent to allow for the development of flow. The particles were released from the start of the bifurcation where flow was solved a significant distance upstream the bifurcation. The finalized mesh consisted of 721678 domain elements, 48668 boundary elements, and 160 edge elements. A boundary condition of flowrate was prescribed at the inlet with a zero-pressure condition at both outlets. The finalized mesh is shown in Fig. S1.
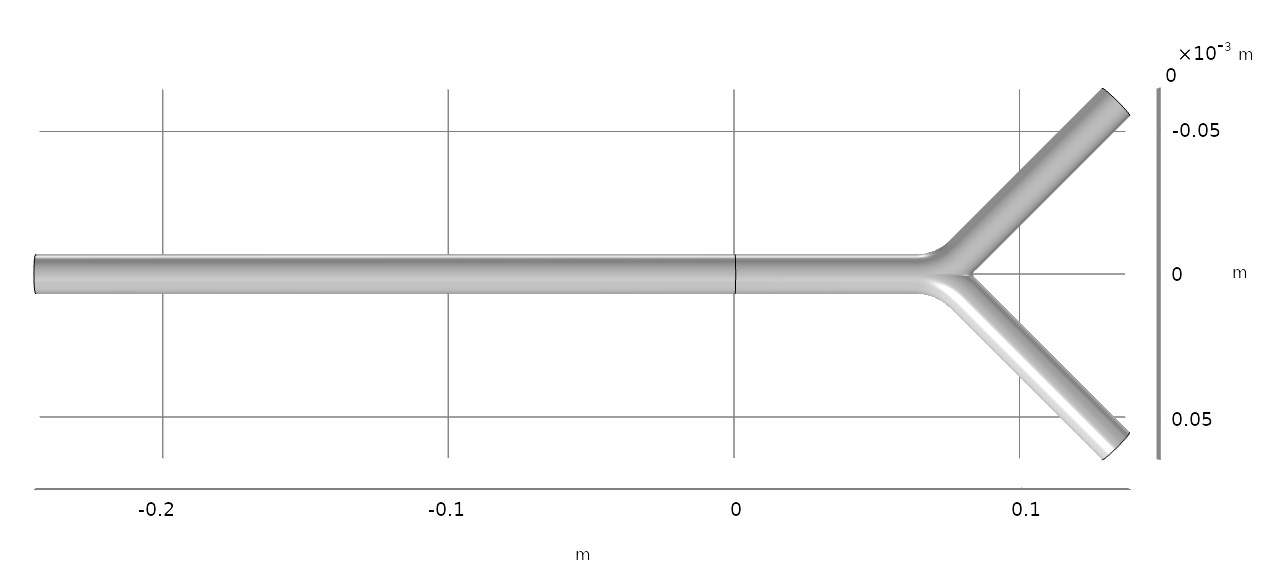
*Figure S1: Geometry used for computational simulations. Two boundary conditions were used in the simulation, one at the left end for flow and a second one at the start of the bifurcation for particle release.*

***Adjacency matrix***

An example of what the adjacency matrix looks like is given in Table S1.

*Supplemental Table S1: The adjacency matrix for a small asymmetric tree*

| State | 11 | 12 | 111 | 112 | 121 | 122 | Captured | Escaped |
| --- | --- | --- | --- | --- | --- | --- | --- | --- |
| 1 | 0.491449 | 0.491449 | 0 | 0 | 0 | 0 | 0.017102 | 0 |
| 11 | 0 | 0 | 0.494359 | 0.494359 | 0 | 0 | 0.011281 | 0 |
| 12 | 0 | 0 | 0 | 0 | 0.496522 | 0.496522 | 0.006957 | 0 |
| 111 | 0 | 0 | 0 | 0 | 0 | 0 | 0.005442 | 0.994558 |
| 112 | 0 | 0 | 0 | 0 | 0 | 0 | 0.010167 | 0.989833 |
| 121 | 0 | 0 | 0 | 0 | 0 | 0 | 0.007054 | 0.992946 |
| 122 | 0 | 0 | 0 | 0 | 0 | 0 | 0.008433 | 0.991567 |
| Captured | 0 | 0 | 0 | 0 | 0 | 0 | 1 | 0 |
| Escaped | 0 | 0 | 0 | 0 | 0 | 0 | 0 | 1 |

Transition probabilities in the second to last and last columns represent the probability that a particle deposits in a given segment and escapes from the last segment, respectively.

***Effect of constriction pattern of flow and deposition***

Several different scenarios of airway constriction were studied to demonstrate the versatility of the Markov chain. First, to study the effects of constricting a single airway at generation 3 to 10% of its original diameter was examined. This represents an extreme case of airway constriction high up on the tree and hence it is not a realistic model of heterogeneous airway disease that occurs for example in asthma. Nevertheless, it is a demonstration of how a single constriction can affect flow distribution and deposition in the region subtended by the constricted segment.


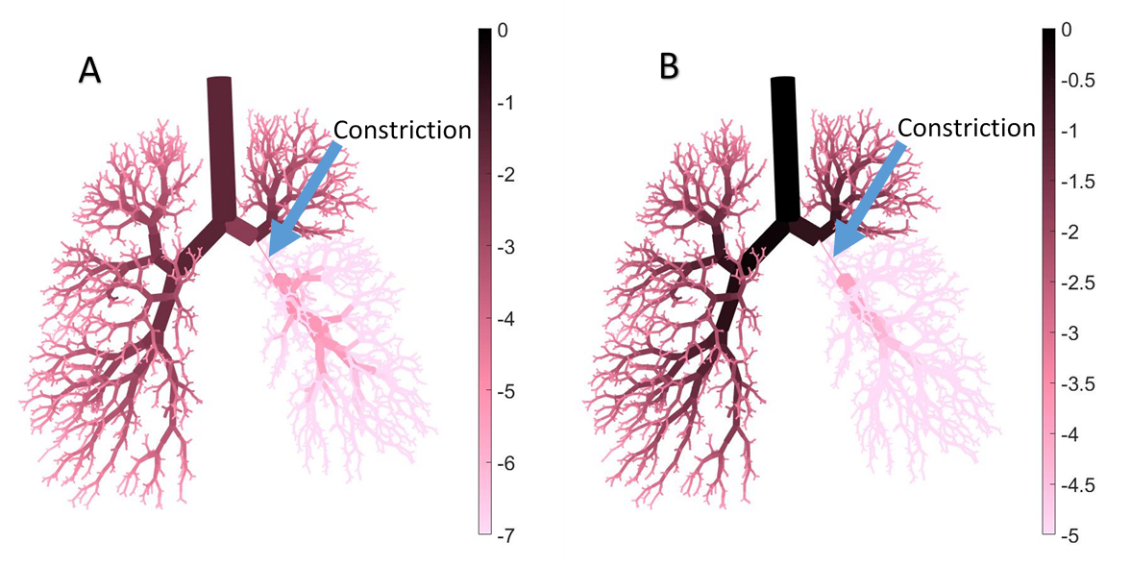


*Figure S2: (A) Flow distribution in the presence of constricting a single segment (blue arrow). (B) Probability of capture for optimal flow policy corresponding to flows in panel A. The colors represent log of flow or log of probability.*

The flow into the subtended tree was drastically reduced (Fig. S2A) due to a diversion of the flow toward a region with smaller resistance, which resulted in virtually no regional deposition therein (Fig. S2B). Maximum flow polices are less efficient during such events and thus an optimal flow policy that deals with the additional risk or too high flow rates becomes more important.

## *Computation of flow in an asymmetric tree*

Flow patterns through a cascade of bifurcations were simulated by solving the 3D Navier-Stokes equations for conservation of momentum and the continuity equation for conservation of mass. The Turbulent Flow k-ε interface in Comsol Multiphysics was used for simulating single-phase flows at high Reynolds numbers. This physics interface is typically used for incompressible and compressible flow for Mach numbers less than 0.3. The conducting region of the geometry used in the Markov model was imported into Comsol Multiphysics. It included the first 67 segments of the asymmetric tree (Fig. S3). A free tetrahedral mesh was created that consisted of 3642724 domain elements, 197024 boundary elements, and 1378 edge elements. An inlet flow boundary was applied at the root of the tree and all other outlet boundary conditions were chosen as zero pressure.


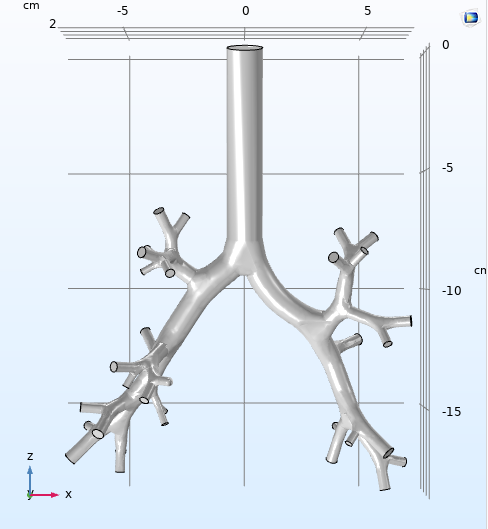


*Figure S3: The geometry used to investigate the influence of inertial flow. This tree was generated using the 3D tree designed by Kitaoka et al^1^ and exported using the software paraview.*

To obtain the total flow through a given segment, first a cross-section perpendicular to the axis of the segment was selected. The velocity field was then integrated over the cross section to obtain flow. Fig. S4 shows how these cross sections were selected.


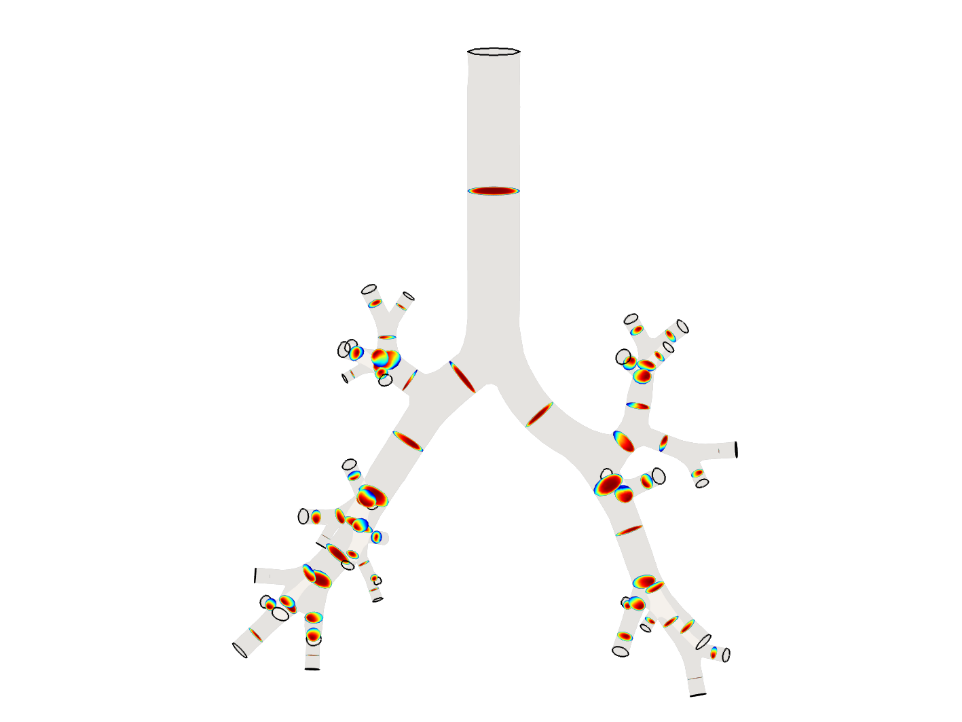


*Figure S4: Cross-sectional planes for each segment of the tree used to calculated flow. A more red color indicates higher velocity.*

Of all the flowrates tested, the following inlet Reynolds numbers were deemed the most physiologically relevant for direct comparison with the resistance-based calculation of flow used in the Markov model: 0.56, 1.78, 5.63, 17.8, 56.3, 121, 261, 563, 1213, 2613, 5630, 12131. Reynolds number, defined here as

$$\begin{aligned} \mathrm{Re}=\frac{\rho uD}{\mu} \end{aligned}$$

where $\rho$ is fluid density, $u$ is the average velocity, *D* is the diameter of the segment and $\mu$ is the dynamic visocity of the fluid.

## *Comparison between CFD and resistance-based flows and deposition*

After the flow rate for each segment of the asymmetric tree was solved, the local flow rates from the CFD were used in the Markov Chain for particle deposition simulations. A representative image of the flow profiles in the asymmetric tree is shown in Fig. S5.


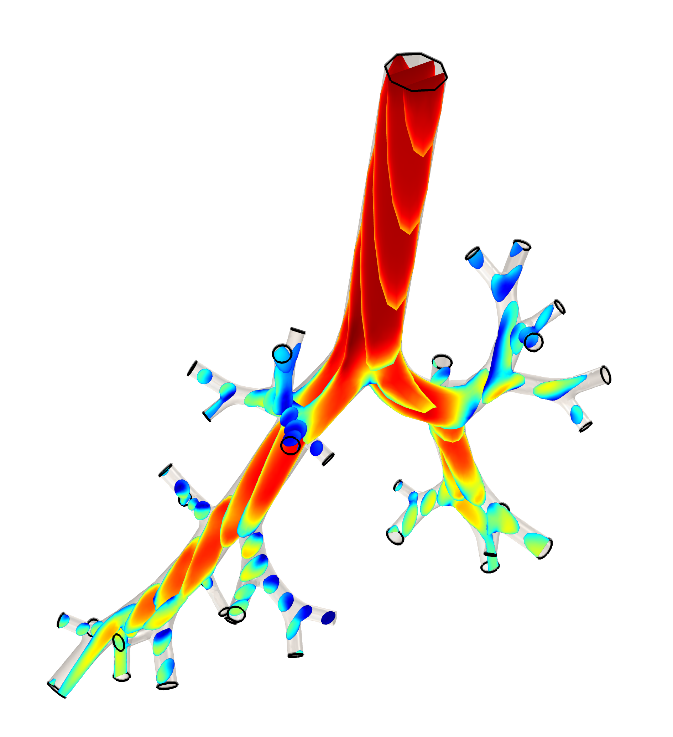

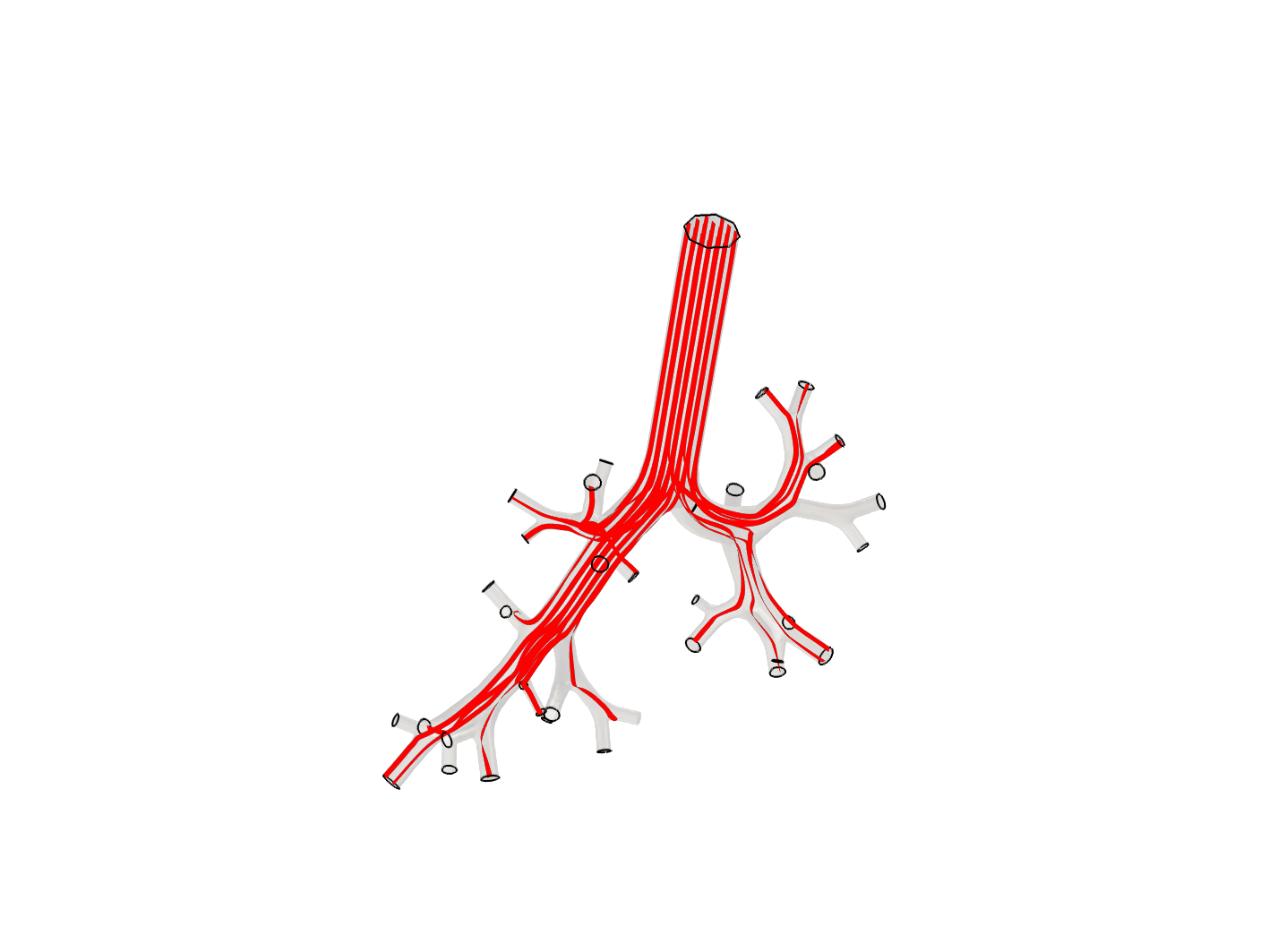


A

B

*Figure S5: Examples of asymmetric flow in an asymmetric structure. A) Velocity contour plots for multiple planes sliced along the xy plane. Higher flow rates correspond to the colors closer to red. B) Streamline plot corresponding to the velocities in panel A.*

A total of 20 different Reynolds numbers were simulated and the flow distribution through the tree was plotted as a function of the inlet Reynolds number. A comparison between the flows predicted by both the downward path resistance model used in the main body of the paper and the CFD model can be seen in Fig S6.

*Figure S6: Error, defined as the fractional difference between CFD predicted flow and resistance-based flow, is plotted as a function of segment diameter. The gray band marks ±20% error. The different colors represent different inlet Reynolds numbers.*

The results in Fig. S6 demonstrate that generally larger errors occurred in segments with a smaller diameter. Next, both flows were used in the Markov chain model to predict deposition probabilities throughout the tree as shown in Fig. S7.


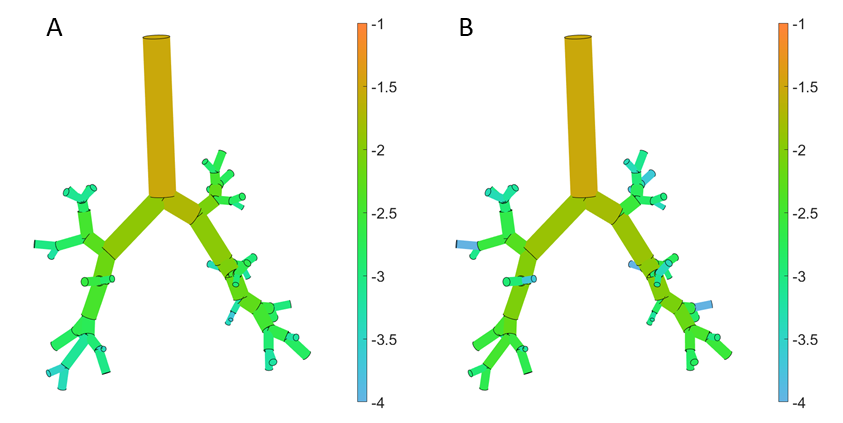


*Figure S7: Steady state probabilities of deposition for the highest error case with Re=12131. The colors correspond to the log of the probability of deposition. Panels A and B correspond to linear resistance-based flows and CFD-based flows, respectively.*

The relationship between the CFD-derived data and the linear resistance-based flow results is important with regards to particle deposition prediction in general. While there does appear to be large discrepancies between the well-established method of predicting flow in the lungs by downward path resistance (Fig. S6), the CFD derived flow’s overall impact on particle deposition is minor (Fig. S7 and Fig. 8 in the main body). From a statistical standpoint, the resistance-based Markov chain provides a reasonable map of particle deposition with relatively small discrepancy compared to the CFD-based Markov chain. This is likely because the probabilistic approach used is not overly sensitive to small perturbations in flow rate. The reason behind this finding is discussed in detail in the main text.

**References**

1. Kitaoka, H., Takaki, R. & Suki, B. A three-dimensional model of the human airway tree. *Journal of Applied Physiology* **87**, 2207–2217 (1999).
